# What methods are used to promote patient and family involvement in healthcare regulation? A multiple case study across four countries

**DOI:** 10.1186/s12913-020-05471-4

**Published:** 2020-07-06

**Authors:** Siri Wiig, Suzanne Rutz, Alan Boyd, Kate Churruca, Sophia Kleefstra, Cecilie Haraldseid-Driftland, Jeffrey Braithwaite, Jane O’Hara, Hester van de Bovenkamp

**Affiliations:** 1 SHARE-Centre for Resilience in Healthcare, Faculty of Health Sciences, University of Stavanger, Stavanger, Norway; 2 Erasmus School of Health Policy and Management, Erasmus University, Rotterdam, the Netherlands; 3 Dutch Health and Youth Care Inspectorate, Utrecht, the Netherlands; 4 Alliance Manchester Business, University of Manchester, Manchester, England; 5 Australian Institute of Health Innovation, Macquarie University, Sydney, Australia; 6 School of Healthcare, University of Leeds, Leeds, England

**Keywords:** Patient involvement, Family involvement, Healthcare regulation, Participation, Decision-making, Norway, England, the Netherlands, Australia

## Abstract

**Background:**

In the regulation of healthcare, the subject of patient and family involvement figures increasingly prominently on the agenda. However, the literature on involving patients and families in regulation is still in its infancy. A systematic analysis of how patient and family involvement in regulation is accomplished across different health systems is lacking. We provide such an overview by mapping and classifying methods of patient and family involvement in regulatory practice in four countries; Norway, England, the Netherlands, and Australia. We thus provide a knowledge base that enables discussions about possible types of involvement, and advantages and difficulties of involvement encountered in practice.

**Methods:**

The research design was a multiple case study of patient and family involvement in regulation in four countries. The authors collected 1) academic literature if available and 2) documents of regulators that describe user involvement. Based on the data collected, the authors from each country completed a pre-agreed template to describe the involvement methods. The following information was extracted and included where available: 1) Method of involvement, 2) Type of regulatory activity, 3) Purpose of involvement, 4) Who is involved and 5) Lessons learnt.

**Results:**

Our mapping of involvement strategies showed a range of methods being used in regulation, which we classified into four categories: individual proactive, individual reactive, collective proactive, and collective reactive methods. Reported advantages included: increased quality of regulation, increased legitimacy, perceived justice for those affected, and empowerment. Difficulties were also reported concerning: how to incorporate the input of users in decisions, the fact that not all users want to be involved, time and costs required, organizational procedures standing in the way of involvement, and dealing with emotions.

**Conclusions:**

Our mapping of user involvement strategies establishes a broad variety of ways to involve patients and families. The four categories can serve as inspiration to regulators in healthcare. The paper shows that stimulating involvement in regulation is a challenging and complex task. The fact that regulators are experimenting with different methods can be viewed positively in this regard.

## Background

In many countries, patient and family involvement is high on the health policy agenda. Efforts to increase involvement can be seen at multiple levels of decision-making: the individual, the organizational and the policy level. The rationale for involving patients and families is two-fold. First, the expectation is that it will lead to better quality decisions across levels and thereby to better quality, and person-centred care. Second, as patients are the ones affected by decisions, they should have the opportunity to influence decisions [1–8].

In the regulation of healthcare, the subject of patient and family involvement also figures increasingly prominently on the agenda [1, 9]. User involvement in regulation can be varied in nature. It can include providing individuals with information about a regulator; mobilizing users and patients as sources of information; and reviewing whether health service providers involve users in service delivery and planning [5, 6]. This means that, on the one hand, involvement of patients and families is incorporated in regulatory standards and expectations directed at healthcare service providers [1, 5, 10–12]. On the other hand, involvement figures increasingly in the work of healthcare regulators themselves [5, 6, 13, 14]. In this paper, our main focus is on involvement initiated by regulators, although we recognize that this is interrelated with regulatory standards addressing involvement in service provision.

There is a growing literature on involving patients and families in their own care, especially in service provision [3, 4]. In comparison, the literature on involving patient and families in regulation remains in its infancy [5]. There are exploratory studies reporting experiments with involvement in different aspects of regulation, such as including patients in inspection teams, theme-based inspections, the investigation of sentinel events, using them as mystery guests and involving patients and families in developing inspection assessment criteria [5, 6, 9, 12, 15–17]. However, despite these examples of specific cases in the literature, a more systematic analysis of how patient and family involvement in regulation is accomplished across different health systems is lacking.

In this paper, we provide such an overview by mapping and classifying methods of patient and family involvement in regulatory practice in four countries; Norway, England, the Netherlands, and Australia. We do so by analyzing the literature and other relevant sources such as the grey literature, websites, and documents of regulators that describe user involvement. By mapping methods of involvement and the use of patient and family experiences in the regulation of healthcare organizations, we provide a knowledge base that enables discussions about possible types of involvement, and advantages and difficulties of involvement, encountered in practice. Such an analysis can help regulators, and also shape the agenda for future research.

The following research questions guided our study:

1. What kind of methods for patient and family involvement in regulatory practice do regulators use in different countries?
2. What are the reported benefits and challenges of involving patients and family members?

The paper proceeds as follows. First, we describe the methods used in our study. Second, in the results section we categorize and describe the methods of involvement that we found in the selected countries and present available evaluations of these. In the discussion, we reflect on our findings and relate them to the literature in order to identify lessons learnt for involvement in regulation, alongside topics for future research.

## Methods

### Research design and case selection

The research design was a multiple case study of patient and family involvement in regulation in four countries. A case was defined as a country and the methods identified for patient and family involvement in organizational regulation of quality and safety in healthcare [18]. The scope of our cases was limited to the regulation and regulatory practice related to organizations such as hospitals, nursing homes, youth care and home care. This means that involvement in regulation of individual healthcare professionals, such as involvement in decisions on disciplining or striking off doctors and nurses from their licenses and registries, was omitted. The regulatory bodies included in our country cases are the Norwegian Board of Health Supervision (NBHS) and County Governors in Norway, the Care Quality Commission (CQC) in England, and the Health and Youth Care Inspectorate (HYCI) in the Netherlands. In Australia, the Australian Commission on Safety and Quality in Health Care (ACSQHC), which developed that country’s accreditation standards, was included as the key stakeholder in this particular regulatory system (see Table 1 for details about the country regulatory contexts and references to official web pages).

**Table 1:** Description of regulatory context per country

Country	Description of regulatory context
**England**	The supervision authority in England is the Care Quality Commission (CQC) [19]. CQC was established in 2009 as an independent non-departmental body, which is at arm’s length from government ministers. The CQC is held accountable to Parliament through the Health and Social Care Select Committee and to the Department of Health and Social Care through quarterly accountability review meetings. The CQC has a unitary board with a majority of non-executive members, which holds public and private meetings. The purpose of the CQC is to make sure health and social care services provide people with safe, effective, compassionate, high-quality care and to encourage care to improve. The CQC regulates the activities of health and social care organizations at almost 50,000 locations, serving the 55 million people in England. The main functions of the CQC are: • Register: Maintain a register of who is legally able to deliver regulated activities. Organizations are required to show they can meet standards of care set out in regulations in order to join the register. Subsequently they must notify the CQC of deaths and other incidents affecting service users, such as deaths not attributable to their illness, injuries, abuse, insufficient staff, and interruption of basic services such as gas and electricity. • Monitor, inspect and rate: Monitor the quality of care by gathering and analyzing data, including from people who use services, providers and other stakeholders. Monitoring informs the inspection of services to make sure they are providing care that is safe, effective, caring, responsive and well-led. Inspection findings are published, including for many services a rating of the quality of care. The CQC protects people by taking enforcement action to address poor care. • Independent voice: Publish reports on major national and regional quality topics, while also highlighting good practice. The CQC does not routinely have a role in investigating adverse incidents or complaints. Healthwatch England, the national consumer champion for users of health and social care services, is a statutory committee of the CQC’s Board. The CQC has a duty in law to take account of the views and experiences of local Healthwatch. (Source: [19])
**The Netherlands**	The regulatory authority in the Netherlands is the Health and Youth Care Inspectorate (HYCI) (Inspectie voor de Gezondheidszorg en Jeugd, IGJ) [20], which is part of the Ministry of Health, Welfare and Sport. This inspectorate regulates and promotes good and safe care. It’s regulatory activities are partly risk-based, thematic and incident based. The work of the inspectorate is based on different national acts focusing on, amongst others, quality of care in different sectors and the governance thereof, individual professionals, complaint procedures, and medication safety. One of the current focus points of the HYCI is person-centred care as it is considered an important condition for providing good and safe care. The HYCI distinguishes between the perspective of the public and the patient. Including both perspectives in its work is high on the regulatory agenda and mentioned specifically in multi-annual policy plans of ‘16-‘19 and ‘20-‘23. A vision document ‘Public and patient’s perspective in regulation’ was also written. The HYCI installed a Coordination Group Public and Patient Perspective in Regulation in May 2018 to collect information, advice and coordinate activities aimed at stimulating the inclusion of the public and patients perspective in regulation. The National Healthcare Report Centre, where citizens can ask questions and report complaints about the quality of care, is part of HYCI. In the Netherlands, healthcare organizations mostly conduct their own investigations in response to incidents. The HYCI requires that they involve patients or family members in these investigations. In addition, the HYCI has conducted many experiments to involve citizens in its inspections (more on this below). The HYCI works together with a number of universities in an academic collaborative where research is conducted into all kinds of aspects of regulatory work. This includes the subject of public/patient participation. (Source: [20])
**Australia**	Regulation of the Australian healthcare system is complex and fragmentary. Responsibilities are shared among a network of national, six state and two territory departments of health, in addition to other government bodies [21]. Accreditation against standards is one of the major strategies for assuring the quality and safety of healthcare organizations. Before the federal government became involved, Australian hospitals were early-adopters of this approach, dating from the 1970s via the Australian Council on Healthcare Standards [22]. Accreditation was initially a voluntary activity. Due to growing scrutiny of hospital adverse events in the 1990s, Australian states passed laws mandating their hospitals take part in accreditation [23]. From 2000, the Council of Australian Governments passed reforms that subsequently established the Australian Commission on Safety and Quality in Health Care (ACSQHC) [24] and made accreditation against the national standards mandatory for all public and private hospitals [23]. However, the accreditation of general practices is still voluntary [25], and national accreditation standards have only recently been made mandatory for government subsidized residential aged care facilities [26]. Unlike other health systems discussed in this paper, in Australia, there is no national regulatory body conducting inspections to ensure health services are delivered safely and according to the law. Rather, the National Safety and Quality Health Service Standards, developed by the ACSQHC, are used by independent organizations who are contracted by healthcare organizations to conduct their accreditation surveys, usually on a 3–4 yearly basis [24]. If any of the standards are ‘not met’, hospitals have up to 3 months to resolve the issue, depending on the risks associated with the issue. At state and territory levels, there are also departments of health, and a range of commissions and divisions that play a role in monitoring and improving the quality and safety of healthcare organizations, such as the Clinical Excellence Commission in New South Wales (Australia’s most populous state). When a standard is not met during an accreditation survey, state and territory health departments are notified and may take other regulatory action or provide support to health services as they address the issue [24]. (Source: [24–26])
**Norway**	The supervision authorities are the Norwegian Board of Health Supervision (NBHS) (the central office), and the Offices of the County Governors (regional offices). The NBHS [27] is a national public institution organized under the Ministry of Health and Care Services. The overall aim of public supervision is to ensure that health and social services are provided according to national acts and regulations. In Norway, there is comprehensive legislation regarding child welfare, health and social services that: • constitute requirements about the services that shall be offered to the population; • constitute requirements about the quality of services; • regulate the work of health care personnel who have authorization; • give users of the services rights, for example, according to the Patients’ Rights Act. Supervision applies to all statutory services, irrespective of whether they are provided by municipalities, private businesses, publicly owned hospitals or health care personnel who run their own practice. Regulatory activities vary from area surveillance, proactive and planned supervision, and reactive event based after adverse events or deficiencies in services. At the level of the counties, supervision is carried out by the Offices of the County Governors. The NBHS has a special Department that can conduct onsite inspections in cases of the most severe adverse events. Most inspection activities are conducted by the Offices of County Governors. (Source: [27])

We aimed to sample a broad range of empirical material from different nations and healthcare systems to illuminate the research questions from several angles [18, 28, 29]. We purposively selected countries with different types of healthcare systems. Notwithstanding this, all are high-income countries where one could expect a developed approach to user involvement at all system levels, including the regulatory level. The rationale for conducting cross-country studies can be for comparative purposes, however the main reason for our case selection was not comparison, but rather to provide a broad-ranging overview. Our case selection enabled access to data on methods of involvement from a variety of contexts. This served as a basis for developing an overview of existing methods and how countries approach user involvement in regulation.

### Data collection and analysis

The data collection was conducted between February and April 2019 according to a template designed by the authors centred on the research questions. The authors based in each country collected 1) academic literature if available and 2) documents of regulators that describe user involvement. These documents included: grey literature, project reports, policy documents, and projects conducted by healthcare regulatory bodies described on their web pages. We felt it important to incorporate the grey literature, as involvement projects in regulation are often not designed as research projects, and therefore not published in peer reviewed journals. We conducted a broad search, as the main aim was to map existing methods of involvement in addition to possible existing evaluative data.

In each country the researchers identified relevant articles and reports based on their expertise of the subject. In addition, they searched academic data bases such as Medline, PubMed, Cinahl, and Google Scholar with search words covering user involvement, patient participation, family involvement, regulation, supervision, inspection, healthcare, quality, quality improvement and patient safety in combination with the names of the countries. We used this approach to identify country specific academic literature. In addition, the Norwegian and the Dutch data collection covered relevant journals in native language where native language search words were used. To identify grey literature and information on the web pages of each country’s regulatory body, the researchers used similar search words as in the literature searches. Moreover, some of the regulatory bodies had gathered information about user involvement and published reports on the topic on their web pages. This helped the researchers to identify relevant projects and reports. In addition, due to the limited literature identified in data bases, some of the research team used Twitter to ask if someone knew of published papers or reports covering the topic of patient and family involvement in healthcare regulation or discussed this question with experts in the field.

The authors from each country completed a pre-agreed template to describe the involvement methods found. The following information was extracted and included if available: 1) Method of involvement, 2) Type of regulatory activity, 3) Purpose of involvement, 4) Who is involved, and 5) Lessons learnt. In addition, researchers from each country provided a short description of their regulatory system, the actors, and their roles and responsibilities in order to provide contextual understanding of the different healthcare systems from which the data was collected.

During the data collection, questions from the researchers were handled by authors SW and HvB, in order to clarify and align the methodological approach across the research team. The discussions were important to ensure a similar approach and trustworthiness of the results. The completed templates were submitted to authors SW and HvB who led the cross-country analysis. During the cross-country analyses, results were synthesized [30, 31], which enabled us to identify existing methods of involvement, commonalities and differences, and clusters of similar types of involvement activities.

In order to find a meaningful way to categorize the involvement methods and to learn from the diversity and similarities, the cross-country synthesis sought to categorize involvement methods inspired by Tritter’s [1] framework from two dimensions: individual vs collective, and proactive vs reactive involvement. This synthesis resulted in four categories of involvement methods, described as 1) individual and proactive 2) individual and reactive, 3) collective and proactive, and 4) collective and reactive (see Tables 2, 3, 4 and 5). The results were categorized as individual if they related to individual patients/user/family experiences with their own care in specific situations. Results were categorized as collective if they related to general aspects where involvement was not related to a specific patient’s own case or treatment, but those involved were expected to represent a group of interests or inform regulatory inspectors on specific topics based on their experiences. We categorized involvement as proactive if the involvement was about collecting information or involving patients/users/families as part of planning future inspections, setting the regulatory agenda or standards, or conducting routine, planned inspections. We characterized results as reactive if the involvement was related to follow up or handling of issues, for example, adverse events, deviances from standards or regulations, or complaints. The results of this synthesis are presented in Tables 2, 3, 4 and 5.^1^ We also analyzed the results in light of the reported benefits and challenges present in the material.

**Table 2:** Norway

**Individual/proactive**	**Collective/proactive**
• Interview with young social care users to inform system audit • Interview with disabled users to inform system audit • Questionnaire to next of kin to inform system audit • Questionnaire to young service users in system audit • Meeting with next of kin to inform system audit • Interviews with service users during system audit • Develop digital tool for communication with children under 13 in child protection services • Including interpreter in system audit of under aged refugees in child protective service institutions	• Patient and user panel established for the regulatory body over time to inform all regulatory activity (national and regional level) • Next of kin as co-investigators in system audit • User involvement in inspection team • Organize seminars with adolescent user organization to inform system audit of child protective services • Develop national regulatory recommendations for user involvement in regulatory practice • Organize co-investigator experience workshop
**Individual/reactive**	**Collective/reactive**
• Meeting between inspectors and next of kin in investigation of deaths • Regulator organizes meeting with patient and healthcare professionals in complaints cases or investigation of adverse events • Regulated right for patient/family to a meeting with service provider after a severe adverse event/death • Regulator contacts/consults with next of kin, informs about the process of investigation of the most severe adverse events conducted at the national level inspectorate investigation unit • Patient and user complaints can be sent to regulator

**Table 3:** England

**Individual/proactive**	**Collective/proactive**
• Patients share experience with services in an online form to CQC • Annual user surveys to collect user experiences of particular service of interest • Inspectors and Experts by Experience speak with children, young people, parents, families, and carers during inspections. • National surveys • CQC commission community groups and charities to collect user experiences of particular pathways or types of care • CQC carries out research and focus groups to collect user experiences of particular pathways or types of care	• Awareness campaign using social media, digital marketing, charity communications channels and other CQC communication channels to encourage patients and families to share their experiences with CQC • Collaboration with national charities to collect information about user experiences via their helplines • Collaboration with Healthwatch to produce guidance for local Healthwatch and Inspectors on working with CQC to promote involvement in reviews, on a general basis, and encourage members to submit user experiences to CQC • Public online community for involvement in health policy and service design – includes both paid vouchers and self-selected groups • Experts-by-experience involvement in thematic reviews (involvement varies depending on topic) • Expert advisory group set up as part of inspection approach - including experts-by-experience members • Establish user panels and advisory groups (children, mental health) • Experts-by-experience used for speaking to people using services, families and organizations, that support them • Experts-by-experience assist in registration, thematic reviews, local systems reviews, co-production, advisory groups, promotion of CQC work, and training of inspectors • Co-production of events by seldom heard communities • “Mystery shoppers” and hidden cameras – CQC has considered this approach but has decided not to conduct covert surveillance • User panels (mental health, children and young) • Commissioned research with users • Data sharing partnership with other websites collecting intelligence from users
**Individual/reactive**	**Collective/reactive**
	• Analyses of complaints and concerns from various sources as part of its risk-based supervision of providers, called “intelligent monitoring”

**Table 4:** The Netherlands

**Individual/proactive**	**Collective/proactive**
• Interview patients or family during planned inspections in all care sectors • Collect experiences from adolescents and other patient groups (such as people living in poverty) in theme based inspections • Interview frail older service users to inform theme based inspection • Search social media to collect information from individual patients as signals in risk based supervision	• User panels on specific regulatory topics • User involvement in multi annual policy plan • Advisory board of children (Children’s Council) • User involvement in designing supervision frameworks • Sharing information about the inspectorate to the public • Use patients as a source of information for planning and conducting theme based inspections • Interview adolescents represented in a youth council as part of theme based inspection • Young peer-inspectors in inspection team to interview young people • Experts-by-experiences (with learning disabilities) involved in the entire inspection process of theme based inspection • Experts-by-experience were trained and involved in inspections in elderly care homes • Mystery-guests with learning disabilities used in review of access of services (experts by experience show up in institutions under-cover and inform inspectors about their experiences) • Mystery guests used in reviews of elderly home care • Search social media to collect information about organizations as signal in risk based supervision • The ratings and reviews of an independent patient rating website are used by inspectors in identifying risks or themes or to prioritize their visits
**Individual/reactive**	**Collective/reactive**
• Patient complaints • Regulatory requirement for involvement of patient/next of kin in incident investigation • Contact next of kin after sentinel events to identify if they have been involved in the required investigation by the healthcare organization	• Inspecting of how healthcare organizations involve patient and next of kin in incident investigation • Aggregated information from complaints on sector and themes is used in risk based supervision for agenda setting and prioritization • Text mining research is performed to find relevant topics in complaints

**Table 5:** Australia

**Individual/proactive**	**Collective/proactive**
• Patient survey at the state level about recent experiences and outcome of care • REACH – Recognize, Engage, Act, Call, Help is on its way: state initiative to empower consumers to ‘speak up for safety’, engage with their nurses or medical team, and request clinical review within 30 min	• Consumers members of clinical governance committees • Involvement of consumers in accreditation standard development • Accreditation standard requires healthcare organizations to partner with consumers in planning, designing, measuring, delivery and evaluation of care • Co-surveyors where consumer are involved in the accreditation process as team members • Consumer involvement in regulation of healthcare research – 2 members are laypersons in all Human Research Ethics committees • Collaborative Pair Program – National program to promote meeting of accreditation standards requiring patient and consumer involvement. Support clinician-patient approaches.
**Individual/reactive**	**Collective/reactive**
• Regulatory requirement for investigation most severe adverse events by a formal root cause analysis (RCA). Interviews with patients and next of kin may be part of the RCA. ◦ Apology to patient and family as part of open disclosure in RCA process • Consumer, patients, families can submit complaints to ombudsman	

All authors are experienced researchers in the field of health services research, regulation, health policy and user involvement. Among the authors, there are also members with a background as regulatory inspectors (SW) or in a current main position as members of an inspectorate (authors SK, SR) in addition to being researchers. This experience in the author team was beneficial in helping to identify important cases, challenges, possible literature, projects, and to suggest possible recommendations of relevance for both the research community and for other regulatory bodies. In England, a senior manager within the CQC was also consulted, to ensure thoroughness in our search, and clarification of the breadth of available involvement methods.

## Results

First, we present a short introduction to the regulatory context of the four countries, before presenting the overall findings relating to involvement methods across countries. We then describe the reported benefits and challenges of involvement based on the evaluations, if available, of experiments included in the study.

### Regulatory context

In Table 1 we provide a brief overview of the regulatory context of Norway, England, the Netherlands, and Australia as a backdrop for this paper. Some countries use the term regulation, while others use supervision or inspection about their role and activity. This is reflected in the context description in Table 1.

### Methods of involvement in regulation across countries

The data synthesis identified a wide variety of methods for user involvement in regulatory practice. In the following, we present the four categories of activities within these. A summary of these results per country can be found in Tables 2, 3, 4 and 5. (To ease readability of Tables 2, 3, 4 and 5 and the four involvement methods categories, we present this text without references. All references are included when we present the reported benefits and difficulties of involvement identified in research studies or in the published grey literature).

### Individual proactive methods of involvement

Individual proactive methods refer to involvement of individuals with the purpose of collaboration and use of information for setting the future regulatory agenda and planning regulatory activities. The results in this category showed that the regulators in Norway, England, and Australia used some kind of patient and user surveys to collect information as part of setting the regulatory agenda and informing inspection activities. National surveys are a way of collecting information about experiences and outcomes of the health services from broad groups of patients and users. We also identified more targeted surveys. In such cases, surveys were designed for specific groups, such as next of kin, and used to inform future system audits and make these more context-specific and relevant for the target group of regulation (e.g., children). Questionnaires were also utilized as part of a regulatory activity to collect information from a broader audience of users in planned inspections.

The regulators also made use of qualitative methods such as individual interviews and meetings with service users. Regulators spoke with children, adolescents, parents, families, next of kin, social and mental healthcare users, disabled users, and frail older service users. We found these methods were part of planned inspections (not initiated due to an adverse event) in Norway, England, and the Netherlands. Regulators collected information based on users’ experiences with the services and used this information to assess if services were provided according to standards and regulation.

The synthesis identified specific efforts among the regulators to involve and get in contact with vulnerable groups with increased risk of not being heard through routine approaches. For example, in some Australian states, surveys are specifically directed at ‘hard to reach’ groups (with low response rates, however). In other countries, hard to reach groups such as young asylum seekers, young people with autism, people with a migrant background, elderly and people with learning disabilities are targeted through qualitative methods. Such methods included the development of digital tools for communication with children under 13, and experimenting with including interpreters in system audits of under aged refugees in child protective service institutions.

### Individual reactive methods of involvement

Individual reactive methods of involvement refer to how regulators involve individuals (patient, user, or next of kin), when they have experienced either an adverse event or filed a complaint about the service provision to the regulator. The Netherlands, Australia and Norway have regulation enabling users, or next of kin, to file complaints to the regulator or an ombudsman, or both. This legal right is one way of involvement in itself, but we also sought to identify if, and how, people were involved in the regulatory process after filing a formal complaint. In the included countries, a complaint can relate to both lack of service provision and being denied a service. In addition, complaints can include reporting experiences of adverse events, near misses, or patient harm. We found examples from Norway where the regulator established meeting arenas between regulatory inspectors, service providers, and users or next of kin. The purpose was to gather the involved parties and through a dialogue-based approach, try to solve each complaint case so it did not proceed to a formal and often long-lasting written information exchange process between the parties before reaching a conclusion.

In this category, we also found methods of involvement in the regulatory investigation of adverse events. A regulatory investigation may be initiated based on patient complaints, or by mandatory reporting of adverse events from the service providers to the regulator. In Norway, results showed several examples of individual involvement methods in the investigation process. In the most severe cases (e.g., deaths), investigated by the NBHS at the national level, the regulator always consults with the family, informs them about the progress and collects their views on the event. Moreover, there were examples at the county level in Norway of regulators organizing formal face-to-face meetings between regulatory inspectors and next of kin to collect information about the adverse event leading to patient death from the next of kin’s perspective. In the Netherlands and Australia, we identified regulatory requirements for service providers to conduct investigations of adverse events, but the regulator does not usually investigate these. However, since both countries require service providers to involve patients and the families in the investigations, this implies that one way of prompting user involvement is by regulating and requiring the service providers themselves to involve patients and family in investigations (as described in the introduction of this paper). In addition, Norway has recently made it mandatory for health service providers to invite patients and users to a meeting after a severe adverse event. In addition, in long-term care, the Dutch inspectorate experiments with checking directly with next of kin whether the service provider has involved them in the investigation process.

### Collective proactive methods of involvement

In collective proactive methods of involvement, the involvement is not so much focused on a patient’s specific case or treatment, but more generally. Users involved are expected to represent a group of interests. The purpose is to inform the future regulatory agenda or specific inspections. The collective and proactive methods of involvement demonstrated a wide repertoire in all the included countries. England and the Netherlands were at the forefront in utilizing a range of approaches. Methods identified in this category related to involvement of user panels and user organizations, campaigns, expert-by-experience and mystery guests, data sharing, and commissioning of research.

Across countries, the most common method of this type is using experts-by-experience, peer-inspectors (in inspection processes) or co-surveyors (in accreditation processes). These methods consider users, patients and family members to be experts on care, which warrants them being a part of planned inspection activities (or accreditation surveys in Australia). The degree of involvement varied from being involved in an inspection planning meeting, to being part of the inspection team as co-investigator on site and in the analysis of results. Experts-by-experience were reported to be involved in thematic reviews and system audits. We identified variations on the expert-by-experience approach in different fields. Examples included adolescents involved in the inspection team to interview adolescents in thematic inspections, co-designing regulatory frameworks, or involvement of people with learning disabilities and next of kin in an investigation process. Moreover, the synthesis uncovered examples of older people being trained and included in the inspection process in elderly care homes. Similarly, the “mystery guest” method was applied in the Netherlands. Mystery guests are a subset of the experts-by-experience approach, where potential service users visited service providers “undercover” and evaluated aspects of the services given. For example, people with learning disabilities assessed the accessibility of services as mystery guests. Another example of the ways in which mystery guests were used, was considering whether the information they received from the service provider would be easy to understand for the group they represented. Based on these experiences the people with learning disabilities, service providers and inspectors discussed the accessibility of the services, what information they wanted to keep and what could be improved. In this particular example, managers were also later involved. The regulator used this information in the assessment of the service provider.

Our synthesis found that user panels and user advisory groups were common in the regulatory bodies across countries. In England, the CQC has user panels to provide input and advice regarding the regulation of children and mental healthcare services. In Norway, the NBHS recently established a user panel at the national level to inform all regulatory activity. Several Norwegian regional regulatory offices have established user panels. The Dutch inspectorate organizes user panels on specific regulatory topics and is investigating the possibility of a structural user advisory board. Furthermore, the Dutch inspectorate involved users in developing their multi annual policy plan ‘20–23’, and the Youth Care department of this inspectorate has been advised by a Children’s Council in 2019. Similar methods that focus on involvement in committees and panels are human research ethic committees and clinical governance committees which are established in Australia. In England, the CQC commissioned research with users.

The regulators also collaborated and organized seminars with user groups, councils, charities, and organizations to collect information and experiences as part of the planning of inspection activities. Some of these activities were co-produced by the user organizations and the regulatory bodies. We also found examples of organizing co-investigator workshops to collect experiences and lessons learnt from the experts-by-experience who had been involved in some kind of regulatory activity.

In this category, we also identified virtual involvement initiatives and campaigns using social media, digital marketing, and other online platforms to encourage patients and families to share their knowledge and experiences with the service providers. Other initiatives online related to disseminating general information to user groups about the regulators’ role and responsibility. In the Netherlands, the ratings and reviews of an independent patient rating website are used by inspectors to identify risks and themes for theme based regulation and to prioritize their visits. The Dutch inspectorate also searches social media for signals from users about the quality of care in the health services they visit. Furthermore, we identified an English initiative where CQC had established a public online community for involvement in health policy and service design. This included both a representative panel and self-selected groups. The CQC additionally took advantage of a data sharing partnership with other websites collecting intelligence from users that is then used by the regulator.

The results show collective user involvement for proactive purposes in Australia in accreditation requiring healthcare organizations to partner with consumers in planning, designing, measuring, delivering and evaluating care. A final example in this category is the national recommendations for user involvement in regulations in Norway. Results from Norway suggest a large emphasis on user involvement in regulation over the previous four-year period (2014–2018), where the NBHS made a strategic effort to improve user involvement in regulation by different means, such as funding innovation projects to test new ways of approaching user involvement in regulation. As a result of the four-year involvement program, the NBHS developed national recommendations for user involvement in regulation. Lessons learnt and strategic actions were summarized and the recommendations related to methods, experiences, and how regulators can and should involve users in their own regulatory activities and their training of inspectors. The recommendations underline patient and user involvement as a core value for health service provision and for regulatory bodies.

### Collective reactive methods of involvement

This category relates to collective involvement after healthcare has failed, in terms of adverse events or complaints, but implies that it is not related to an individual’s own specific case. The collective reactive methods repertoire was limited in all countries. Nevertheless, we note some direct examples whereby the Dutch inspectorate aggregated the information from complaints collected at the National Healthcare Report Centre by sector and by theme. Also, the Dutch inspectorate performed an explorative pilot on text mining the content of the complaints for relevant topics. Similarly, in England, the CQC analyses complaints and concerns from various sources. This information is used as a signal in risk-based regulation for agenda setting and prioritization in both countries.

### Reported benefits of involvement

The above synthesis shows the varied nature of user participation in regulation in terms of the type of regulation and the type of involvement. Not all the initiatives mentioned above have been evaluated. Evaluation practices differ between countries. Comparatively, most practices have been evaluated in the Netherlands where the inspectorate works with researchers in an academic collaborative. The practices that have been evaluated do offer important insights into the main reported benefits and challenges of involvement practice.

First, the primary espoused reason for involvement is improving regulatory work, and by consequence improving the quality and safety of care [5, 16, 17, 32–47]. In this view, patients and families are seen as an additional information source. In case of incident investigations for example, patients and families are able to put incidents into a broader perspective and offer a more holistic understanding of what happened [13, 14, 16, 32, 33, 48]. The espoused reasons are similar for the input they provide during thematic inspections [17]. Similarly, experts-by-experience can provide important knowledge as they are more able to tap into experiences of patients, especially in the case of sensitive subjects such as alcohol misuse amongst adolescents [12, 15, 49, 50]. Experts-by-experience and related methods also offer another view on quality of care by focusing more on relations and ‘softer’ aspects of service provision (such as quality of the food, time spent outside, decorative aspects of living facilities) rather than concentrating on safety [15, 51–54]. Also, by analyzing reviews written on an independent patient rating website and social media posts, the regulator may identify risks from the patient’s perspective [35–37, 55–58].

Second, involvement is said to legitimize decision-making of the regulator by using information gathered from patients and the family in the regulatory assessment, but also by co-producing inspection criteria [5, 6, 54]. This relates to the goal of democratic decision-making. For example, when patients and the families support the findings of the regulator this is added to regulatory reports [6, 12, 54]. Patient and family involvement is also related to being transparent about the work of the regulator, and connecting with patients or the public more generally improves the image of, or trust in, the regulator [5, 13, 14, 48]. This argument is not only used to support the active involvement of patients and families, but also to send information through, for example social media, about the work of the regulator to the public [55, 56].

Third, involvement is a way of achieving justice for those affected. This especially applies to the involvement of patients and families during incident investigations as shown in Norway, the Netherlands and Australia. Here their involvement is a way of regaining trust and restoring the therapeutic relationship, including the opportunity to apologize [13, 14, 32, 48]. Moreover, involvement allows for the provision of information to the patient and family, as well as space to share emotions and provide aftercare [13, 14, 16, 32, 33, 40, 48, 59, 60]. Also, some reports mentioned that through involvement escalation of issues to legal claims could be avoided [16, 40]. The need for justice for those affected by poor care can also be found in relation to other methods. For example, in a case of conducting interviews with the elderly, it was shown that participants appreciated the genuine interest in their perspective and felt it was positive to be heard [61]. This was also found in relation to involvement of next of kin in the regulatory investigation of adverse event when patients had died. Next of kin expected to be involved and evaluation showed that involvement could have a therapeutic effect [13]. The regulatory inspectors in this case found involvement to be in accordance with overall political expectation [14].

Fourth, involvement can be a way for users to empower themselves and learn new skills [12, 17, 41, 49, 50, 54]. For example, experts-by-experience learn a lot about a specific subject (e.g., elderly care, alcohol misuse) and the work of the inspectorate [17, 49, 52].

### Reported difficulties concerning participation

Along with the possible benefits reported above, the results also show a number of difficulties experienced by regulators when putting involvement into practice. First, it can prove difficult to incorporate the input of patients and families into the decisions or reports of the regulator [6, 15, 16, 32, 40, 48]. This has partly to do with the perceived lack of legitimacy of patient or family input. It was regularly argued that patients lack the necessary knowledge to contribute, and less weight is given to their input than, for instance, the input of professionals (e.g., in case of incident investigations, theme based inspections or experts-by-experience) [6, 12–14, 16, 32, 35, 48, 51]. As described earlier, the main argument for involvement is that patients and families provide additional information. However, in cases of conflicting input (e.g., different views on what an incident is, what should be the focus of the investigation or what should be considered good quality care) the contribution of patients and family appears to be difficult to incorporate [6, 16, 48]. As a result, there is a danger that involvement becomes tokenistic [44, 45]. A recent example from Dutch research tackles this conflict by making the client perspective the starting point for regulation. Inspectors followed the client’s perspective and judgements throughout the whole inspection process [54]. However, possible conflicts are likely to persist, and the question remains “how will inspectors make informed judgements in such cases in order to do justice to the complexities of regulatory practice”?

Second, not all patients or family members want to be involved or are easy to involve. For example, in case of incident investigations it has been reported that participation is too burdensome as the incident can have a large impact on patients and families [16, 32, 33, 48]. In other cases, it is difficult to do justice to the diversity of patients, as some groups are more inclined to participate than others. This leads to questions concerning the representativeness of those involved and for whom they can speak [5, 48]. This is also an important difficulty attached to professionalization attempts of participants. For example, experts-by-experience are often trained for their task. This may ultimately diminish the value of the authentic perspective that they can provide [12].

This leads us to the third difficulty we identified, that participation involves time and costs [5, 13, 14, 33, 34, 40, 48, 54]. This is especially true for practices directed at so-called ‘hard to reach’ groups because the regulator must expend significant time and effort to come into contact with them, and attune participation to their needs. User participation in general is also expensive. For example, the CQC’s Experts By Experience program has cost an average of £4 million annually in recent years (CQC news). Inspectors in a pilot project in Norway sometimes found it time consuming to involve next of kin due to difficulties in scheduling meetings and the need for additional follow up contacts to collect new information from, for example, actors newly implicated in contributing to the cause of an adverse event. Inspectors did not recommend continuing this involvement method unless it was compensated properly in the work schedule and incorporated in the organization [48].

Fourth, organizational procedures can stand in the way of involvement. This includes the language used by inspectors, and is also due to certain protocols [6, 16, 32]. For example, incident investigations need to happen in a specific timeframe, the deadline of which can be too soon for the patients or family to be able to participate [16, 32, 33]. Also, the regulatory context can prohibit taking the input of patients on board. The case of youth involvement in a thematic inspection on care for children growing up poor by the Dutch inspectorate is a case in point. The interviewed youths stated that they felt their privacy was very important and therefore professionals should not share information about them with each other. In this case part of this conflict was influenced by the regulatory context as policy makers and regulators put much emphasis on sharing information in response to fatal incidents, which were analyzed as resulting from a lack of sharing information [6].

A final difficulty was identified as dealing with the emotions of those involved. This not only applies to the emotions of patients and family members, but of the professionals and regulators themselves, who can also be affected by participation. Emotions can be felt especially keenly during incident investigations [13, 14, 16, 48]. For the next of kin who have lost a close relative, it can be a considerable mental strain to be part of the entire investigation process when they are grieving and sometimes traumatized. The investigation process can repeatedly remind them of the event leading to the death. However, the interpersonal skills of the inspectors may help to reduce this emotional burden. From the inspectors’ point of view, it can be emotionally challenging to involve next of kin in investigation meetings, because the inspectors can worry about attending beforehand and might continue to dwell on things after the meeting, especially in severe cases. Some inspectors have a legal professional background where they were not trained for face-to-face meetings with people in grief, as inspectors with a healthcare background may have been. At the same time, inspectors report that it is a positive experience to offer support to the next of kin and clarify any misunderstandings or questions [13, 14, 48]. In other cases emotions can play a role as, for example, a project involving mystery guests, where civil servants providing services to people with learning disabilities reported feeling left out as their perspective was not sufficiently taken into account [54].

## Discussion

### Unpacking the landscape of user involvement in regulation

As studies into patient and family involvement in regulation are scarce (e.g., [5, 9]) one could question whether regulatory bodies have been part of the general trend in healthcare to involve patients and family members in decision-making. Our study reveals that regulators are very much part of this development. They are in fact experimenting with a wide variety of user, patient, and family involvement methods. Although the context, way of organizing and regulatory environment varied by country, and some regulators are experimenting more intensively with involvement than others, we saw a variety of methods in each of our cases. Regulators used methods in all four categories identified in the paper.

The study shows that the benefits of involvement are multi-faceted. Reported advantages from the published empirical research include for example increased quality of regulatory practice – because it is informed by the unique experiences and insight of people who use care services. Moreover, patient and family involvement increased legitimacy, empowerment, and contributed to justice done to those affected by adverse events (see also [13, 14, 54]). We thus found that involvement can serve multiple purposes – such as in strengthening the quality of regulatory practice, and being beneficial for the users, patients and families involved [13, 14]. In addition, involvement can be used as an instrument to prevent further escalation of problems, through formal regulatory investigation processes or legal claims [11, 16].

Despite this reported added value, our study also identified challenges of user involvement in regulation. It is not easy to develop a regulatory culture where involvement is meant to be integrated into work practice. Also, it is not clear how to use information from experts-by-experience. This is especially important if involvement is conducted to comply with political expectations and not as a way of improving the quality of regulatory activities [40, 62]. In many cases, information provided by users introduces an additional, and different, perspective, but this also means that it can clash with the perspective of, for instance, professionals or regulators [6, 16, 48, 63].

Our findings suggest that the input from users has sometimes been put aside by questioning its legitimacy. If this happens, it equates to epistemic injustice [64, 65] – especially if the knowledge base of users, patients, and family members is considered of less value than those of other actors. This can clearly occur if these extra perspectives do not fit with longstanding regulatory procedures, or with views held strongly by professionals, managers and regulators [66–68].

Other challenges include that not all patients and family members want to be involved, involvement costs time and money, and it can pose an emotional burden on those involved (see also [13, 14, 69]). The emotional burden is especially pertinent to the individual reactive involvement category discussed above [5, 13, 14]. If user involvement in regulation is a question of morality or logic [8] then how regulators deal with the challenge of emotionality, or epistemic injustice [64], should be further investigated to understand the rationale and experiences of regulatory bodies.

There are a number of lessons for regulatory practice. A clear lesson is to target involvement activities and to take advantage of existing opportunities [5, 6, 54]. Since participation is time-consuming for all involved, it is important to consider for which situations it is most important, and to identify the best ways to go about it. The case of using mystery guests with learning disabilities in the Netherlands is a good example in this regard [17, 54]. The mystery guests were asked to assess the access to municipal services for people with learning disabilities, an assessment which can only be adequately made through experience.

There are also ways to include users, patient, and family perspectives based on existing information which can be exploited [5]. Examples of such involvement are 1) to use social media as a way to trace problems in healthcare, 2) to aggregate information from patient complaints, or 3) to perform text mining from patient complaints to search for new types of risks and topics for future inspections [35–38, 57, 58, 70–72]. While uniquely representing quality of care from a patient’s point of view, however, this requires further reflection on methodological issues such as the reliability and validity of these (public) resources.

Another lesson is that involvement needs an embedding strategy. Regulators should think about how to support involvement in their activities, including valuing the financial and time investments [69]. There is also a need to train inspectors such that they can reflect on how to judge different perspectives on quality and how to deal with the emotional burden that can accompany involved users [13, 14]. Good judgement on how and when to incorporate the input of patients is also needed, if that input is to be productive. This might warrant changes in procedures and regulatory frameworks as patients and regulators can have different values and perspectives (e.g., what counts as an incident, how quality of care should be judged) [6, 16, 54, 63]. In this regard, it should be emphasized that participation on its own does not necessarily lead to increased patient-centeredness or learning [2, 32]. An important question to reflect on is how to conduct training of users, patients, family members, and inspectors to succeed with involvement in regulation [12]. The danger is that such training may lead to the professionalization of patient input, which can distance the participants from their experiences as patients [62]. This is problematic because tapping into these experiences is often considered the most important reason for involvement [2, 73].

Finally, it is important to make participation user-friendly and to ensure inclusiveness. In England, much effort is used in making forms and websites accessible, and in building relationships and trust with people from vulnerable groups. Our findings indicate that involving ‘hard to reach’ groups might be challenging, but it can be done [74]. All-in-all, further investigation is needed to evaluate targeted involvement methods of hard to reach groups.

### Strengths and limitations

Several researchers participated in the data collection, which made it possible to include information from various countries and sources. This, however, may also have created variation in data sources. This limitation was moderated by using the template and having numerous discussions between authors.

The regulatory regime is complex [74] and the available data in each country varied, especially in terms of the number of published empirical studies. Most research on involvement in regulation has been conducted in the Dutch setting. We cannot be sure that this is a complete mapping, since involvement methods can be used in regulatory practice without being published or even identified as ‘regulatory practice’. We used data from different sources, such as internet websites, evaluation reports, and peer reviewed papers. This broad approach was important in providing an overview of methods available, in addition to documenting possible advantages and disadvantages and lessons learnt that are relevant to other regulators.

We categorized methods according to the proactive-reactive and individual-collective dimensions inspired by Tritter’s [1] published framework. We used the dimensions as a heuristic tool to gain insight into the variety of methods. Tritter’s [1] original work on reactive and proactive involvement distinguished between whether participation is responding to a pre-existing agenda, in our case set by a regulator (reactive), or if the participants are helping to shape it (proactive). We have added to this conceptualization by including: 1) if participants have experienced an adverse event and responded according to the regulators’ procedures for follow up (reactive), or 2) if the participants have not experienced an adverse event, but provide information to the regulator based on their experiences, to influence future regulatory activities (proactive). On the one hand, this illustrates the strengths, showing that with a few changes, Tritter’s [1] framework may be broadly applicable, including in the regulatory context. On the other hand, the slight deviance from the original version could be considered a limitation.

We do not know whether the diversity we found in methods relates to country characteristics, regulatory regimes or other factors. Further studies might continue investigations along these lines. Examining a number of different countries with diverse regulation systems was a strength as it enabled us to identify a variety of ways of involving users. However, it also made it difficult to determine to what extent differences in user involvement were the result of the different countries’ regulatory systems.

## Conclusion

Our mapping of user involvement methods brought into focus a broad variety of methods. These can serve as inspiration to regulators in healthcare. Based on our mapping exercise we suggest that future regulatory practice continues to develop and pilot new types of user involvement methods including individual and collective, and proactive and reactive activities. The paper shows that making involvement in regulation successful is a challenging and complex task. Our findings suggest that it is not easy to reach the goals of increased involvement and democratic decision-making. The fact that regulators are experimenting with different methods can be valued positively in this regard. As a result, regulators have room to innovate and evaluate work involving new kinds of groups (e.g., older people, youths, people with learning disabilities); new topics and areas of inspections (e.g., social care, elderly care, care transition); different degrees of involvement (e.g., involved in a meeting vs part of inspection team during inspection processes); and new ways of engaging with users and service provision (e.g., mystery guests). Experimenting with these methods means that lessons can be drawn to improve involvement practices, such as how the perspective of users can be incorporated in the judgements that regulators make or in accessing hard to reach groups. For this, further research into these programs is recommended.

Most research is seen in the Dutch context. However, these studies suggest that the promotion of collaboration between regulatory bodies and research groups to build a network for research-based-regulation and regulation-based-research, may help stimulate better research, regulation, and educational programs for future regulators. More international collaborations between researchers and inspectorates could provide further impetus, for instance via the European Partnership for Supervisory Organisations in Health Services and Social Care (EPSO) or the Supervision and regulation Innovation Network for Care (SINC) that inspectorates from various countries established recently. Further development of similar collaboration arenas should be stimulated. As the issue of user involvement is high on the agenda of many regulators, it underscores the importance of this collaborative research agenda.

## Data Availability

All data are publicly available online and cited in the manuscript and in the reference list.

## Abbreviations

ACSQHC: Australian Commission on Safety and Quality in Health Care
CQC: Care Quality Commission
EPSO: European Partnership for Supervisory Organisations in Health Services and Social Care
HYCI: Dutch Health and Youth Care Inspectorate
NBHS: Norwegian Board of Health Supervision
RCA: Root Cause Analysis
SINC: Supervision and regulation Innovation Network for Care

## Footnotes

1 The Tables 2, 3, 4 and 5 may be empty or sparse because of how regulation and governance of healthcare is organized. For example, in England, one reactive category in the country table is empty because CQC does not routinely have a role in investigating adverse incidents, and most complaints are handled by other organizations. We would also note that analyses of complaints and incident reports occur at various levels, but not necessarily as a direct response or mechanism of regulatory agencies, or with patients and families involved.
